# The Role of the Ophthalmic Genetics Multidisciplinary Team in the Management of Inherited Retinal Degenerations—A Case-Based Review

**DOI:** 10.3390/life14010107

**Published:** 2024-01-09

**Authors:** Marcus P. Conway, Kirk A. J. Stephenson, Julia Zhu, Adrian Dockery, Tomas Burke, Jacqueline Turner, Francois Thai Le, James J. O’Byrne, David J. Keegan

**Affiliations:** 1 Mater Clinical Ophthalmic Genetics Unit, Mater Misericordiae University Hospital, D07 AX57 Dublin, Irelanddkeegan@mater.ie (D.J.K.); 2 Eye Clinic Liasson Officer, Vision Ireland, Mater Misericordiae University Hospital, D07 AX57 Dublin, Ireland; francois.thaile@vi.ie

**Keywords:** inherited retinal degenerations, ophthalmic genetics, syndromic retinopathy, multidisciplinary team

## Abstract

(1) Background: Inherited retinal degenertions are rare conditions which may have a dramatic impact on the daily life of those affected and how they interact with their environment. Coordination of clinical services via an ophthalmic genetics multidisciplinary team (OG-MDT) allows better efficiency of time and resources to reach diagnoses and facilitate patient needs. (2) Methods: This clinical case series was conducted by a retrospective review of patient records for patients enrolled in the Target 5000 programme and managed by the OG-MDT, at the Mater Hospital Dublin, Ireland (*n* = 865) (3) Results: Herein we describe clinical cases and how the use of the OG-MDT optimizes care for isolated and syndromic IRD pedigrees. (4) Conclusions: this paper demonstrates the benefits of an OG-MDT to patients with IRDs resulting in the holistic resolution of complex and syndromic cases. Furthermore, we demonstrate that this format can be adopted/developed by similar centres around the world, bringing with it the myriad benefits.

## 1. Introduction

Inherited retinal degenerations (IRD) are a heterogeneous group of genetic retinal pathologies and have been recognised as the leading cause of blindness in the working age population in western countries, as well as an important global cause of childhood blindness [1,2,3]. In addition the variable visual impacts of IRDs, many patients also have retinal degeneration as part of a syndromic condition with multi-system involvement (e.g., deafness in Usher syndrome or neurological manifestations in spinocerebellar ataxia) [4]. The now well documented burden of disease highlights the importance of appropriate personalized management, care, and research into treatments for these patients [5,6,7,8,9,10].

As there is considerable phenotypic overlap amongst IRDs, an increasing emphasis has been placed on genetic characterisation to refine a more accurate diagnosis (e.g., a diagnosis of ‘retinitis pigmentosa’ may be honed to ‘dominant *PRPF31*-retinopathy’ after confirmatory genotyping). A genetic diagnosis also serves to provide patients with guidance regarding prognosis, mode of inheritance for family planning and may open access to novel genetic therapies in the era of personalised medicine [11,12]. The use of genetic testing for patients with suspected IRD has been promoted by the European Reference Network for Rare Eye Diseases (ERN-EYE), acknowledging that genomic testing can improve diagnosis and management of IRD. The ERN-EYE was created ‘to improve diagnosis and treatment of complex or rare medical conditions that require specialised treatment, knowledge and resources’ [13]. It was observed that critical gaps remain in genomic testing in both small and large European countries. One such barrier to effective genomic testing in IRDs is access to healthcare specialists and genetic counsellors who specialise in ophthalmic genetics allowing interpretation of results [13].

As previously alluded to, the complexity of IRDs result in the need for coherent, collaborative multidisciplinary input to achieve optimal patient outcomes. With this in mind, it should be noted that fragmented and poorly optimised care for IRD patients, delivered by various healthcare providers across multiple disparate facilities, represents an inefficient use of resources, delays accurate diagnosis, may introduce miscommunication placing further strain on patients and their families [5,9,12]. In our experience, a structured ophthalmic genetics multidisciplinary team (OG-MDT) approach with regular hybrid meetings (i.e., in-person and remote) has facilitated optimal patient care with best use of available resources, while garnering required expert multisystem opinions. Providers experienced in IRD care (i.e., ophthalmologists/retinal specialists, clinical and molecular geneticists, genetic counsellors) and subspecialists as appropriate (e.g., nephrology, otology, neurology, paediatricians) can be coordinated with set goals and timelines. This concentration of expertise amalgamates expert opinions based on harmonized clinical and genetic diagnoses to reach consensus management decisions [12]. The role of the genetic counsellor, in particular, has been identified as crucial [14]. Patients provided with genetic counselling have an enhanced understanding of their condition, inheritance patterns, medical terminology and are often provided with personalised educational documentation leading to greater patient satisfaction with the MDT service [6,9].

Both clinical (e.g., multimodal imaging) and genetic diagnostic capacity is ever growing. The cost of genomic sequencing has dropped significantly over the past decade, allowing greater testing capacity, and has contributed to the growing utilisation of whole exome sequencing (WES) and whole genome sequencing (WGS), versus more limited panel-based testing [7,13]. However, the coordination and interpretation of these data is best served in a setting where genetic and clinical data is rationalized together. Additionally, local MDTs may link into international networks (such as the ERN-EYE clinical patient management system) for second opinions and collation of data (e.g., novel variant comparison) with genetic eye disease experts internationally.

With this in mind, the Target 5000 programme was established in 2009, to provide an all-Ireland approach to the management of IRD [10]. This took the form of specialist IRD clinics, providing detailed phenotyping, followed by genomic testing through both research and accredited laboratories. A core aspect of the Target 5000 working group at the Mater Hospital is the OG-MDT. Team members consist of consultant ophthalmologists with a specialist interest in IRD, trainee ophthalmologists, a clinical geneticist, clinical scientists, a genetic counsellor and a social liaison/eye clinic liaison officer. Together this team allows reconciliation of genotype with phenotype, particularly in complex cases, and facilitates the creation of individualised care plans [10].

Herein, we use clinical vignettes to demonstrate the power of the OG-MDT.

## 2. Materials and Methods

**Figure 1:**
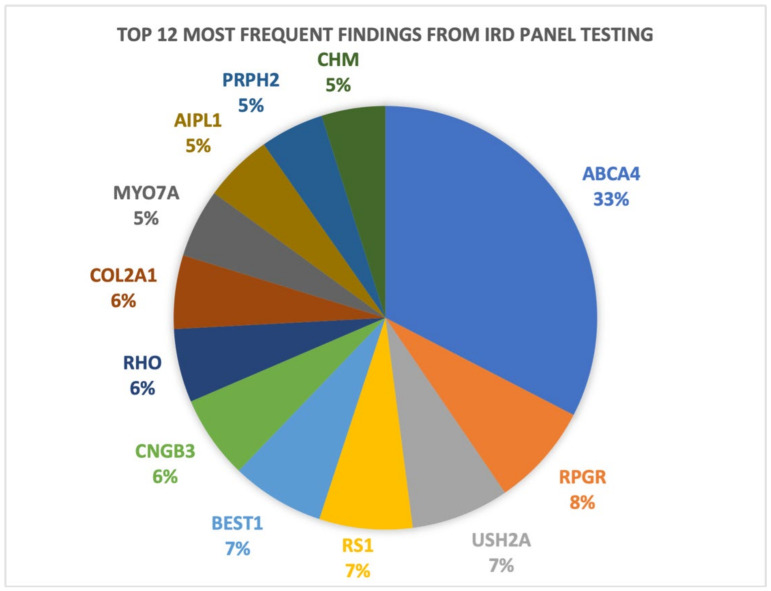
A graphical representation of the 12 most frequent genetic results obtained as a result of genomic testing in patients suspected to have an IRD at the Mater Hospital Target 5000 clinic.

**Figure 2:**
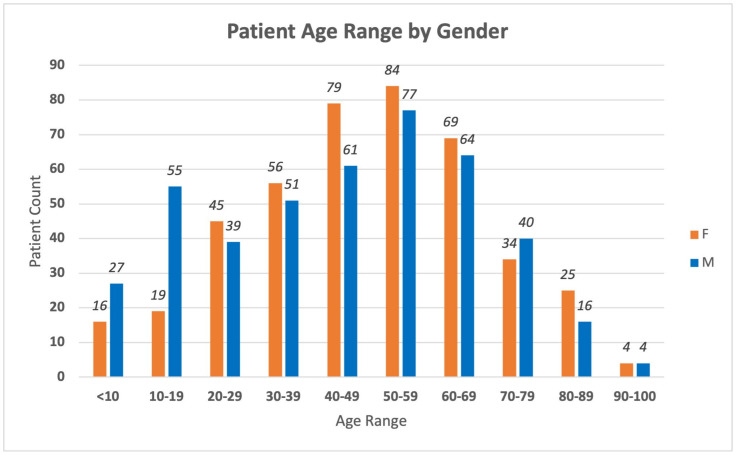
A graphical breakdown of the Target 5000 patient database demographics by age and gender. Total patients (*n* = 865). Unaffected family members and those with carrier status (*n* = 159).

This clinical case series was conducted by a retrospective review of patient records for patients enrolled in the Target 5000 programme and managed by the OG-MDT, at the Mater Hospital Dublin, Ireland (*n* = 865), with unaffected family members and those with carrier status representing 18% (*n* = 159). For a breakdown of the Target 5000 genetic testing results and patient demographics see Figure 1 and Figure 2. The overall diagnostic yield for symptomatic patients who have undergone genetic testing in the programme is 84.1%, greater than our previously reported diagnostic yield of 70% [15]. This figure includes both results from IRD panel testing, and those phenotypes resolved with further genetic testing, e.g., WES and array comparative genomic hybridisation (array CGH). All patients enrolled in Target 5000 provided informed consent granting permission to publish anonymized findings.

Clinical analysis included detailed medical and family history, visual acuity (VA), colour vision assessment, visual field testing, dilated slit lamp biomicroscopy, intraocular pressure (IOP) assessment and multimodal imaging. Along with the family history a multi-generation pedigree was expanded upon by a genetic counsellor, who also provided post-genotyping genetic counselling after discussion at the OG-MDT.

Diagnostic imaging and investigation, where required, consisted of; optical coherence tomography (OCT) (Cirrus 5000, Carl Zeiss MediTec, Dublin, CA, USA), Optos ‘California’ ultra-widefield colour imaging and autofluorescence (AF, Optos plc, Dunfermline, UK), visual electrodiagnostic testing full-field Ganzfeld electroretinogram (Metrovision, Perenchies, France), optical biometry (IOLMaster 500, Carl Zeiss MediTec, Dublin, CA, USA). OCT images were annotated as follows: red arrow—outer nuclear layer (ONL), yellow arrow—retinal pigment epithelium (RPE), white arrows—cystoid macular lesions (CML). AF images were annotated as follows: blue arrow—optic disc drusen, white arrow—macular atrophy/hypoautofluorescene and green arrow—peripheral atrophy/hypoautofluorescene.

Due to the funding model, genetic testing in the Target 5000 programme initially took the form of research grade IRD panel testing, as highlighted in case one, supported by variant confirmation by the Manchester Centre for Genomic Medicine. Public funding was arranged to facilitate genetic testing through Blueprint Genetics (Espoo, Finland) using the retinal dystrophy plus panel (351 genes with mitochondrial genome). Candidate variants are filtered by the in-house molecular genetics team and each case is discussed at the OG-MDT to ensure correlation of phenotype with candidate genotype.

The outcomes of OG-MDT are as follows:

1. Needs further molecular genetic assessment.
2. Needs further or repeat deep clinical phenotyping (ocular and/or systemic).
3. Confirmed match of phenotype and causative (class IV or V variant) genotype.
4. Decision regarding novel (e.g., gene therapy, clinical trial) or established (e.g., cataract surgery, refraction) treatment.

Herein, we illustrate cases of pedigrees with a suspected diagnosis of IRD and the role of the OG-MDT in the diagnostic and therapeutic process.

## 3. Results/Case Reports: (Table 1)

### 3.1. Case 1: Non-Syndromic Disease [MFRP-Associated Retinopathy with Nanophthalmos]

A 56-year-old female was referred to the OG-MDT by her ophthalmologist, with a background of high hyperopia and an historic clinical diagnosis of RP. At initial diagnosis the patient underwent bilateral YAG laser peripheral iridotomies (PI) for acute angle closure associated with high hyperopia and short axial length. She reported onset of nyctalopia, progressive VF constriction and reduced VA from her 4th decade. She reported no relevant medical history. She had seven unaffected siblings and her parents were non-consanguineous. She reported a maternal history of un-investigated nyctalopia, and a maternal uncle with RP, without genetic confirmation.

**Table 1:** Summary of Representative Cases. CG—clinical geneticist; CRD—cone-rod dystrophy; CS—colorectal surgery; Endo—endocrinology; EOSRD—early-onset severe retinal dystrophy; Ex—extinguished; GC—genetic counsellor; Neph—nephrology; NLP—no light perception; Ophth—ophthalmologist, PN—paediatric neurology; RD—retinal dystrophy; WES—whole exome sequencing.

Case	Age & Gender	VA OD	VA OS	ffERG	Clinical Diagnosis	Genetic Diagnosis	Inheritance & Gene	Test Used	MDT Personnel Involved
1	23F	6/15 (20/50)	6/48 (20/160)	Ex	RP	Lynch Syndrome	AD *PMS2*	250 RD Panel, trio WES	Ophth, GC, CG, CS
2	46M	6/30 (20/100)	6/38 (20/125)	CRD	RP, diabetes, obesity	Bardet-Bield Syndrome	AR *BBS1*	351 RD Panel	Ophth, GC, CG, Endo
3	32F	1/60 (20/1200)	NLP	Ex	EOSRD	Knobloch Syndrome	AR *COL18A1*	351 RD Panel	Ophth, GC, CG, Neph
4	56F	6/36 (20/120)	6/60 (20/200)	Ex	RP, nanophthalmos	MFRP-retinopathy/nanophthalmos	AR *MFRP*	351 RD Panel	Ophth, GC, CG
5	6F	6/190 (20/630)	6/190 (20/630)	CRD	EOSRD	Batten Disease	AR *CLN3*	351 RD Panel	Ophth, GC, CG, PN

**Figure 3:**
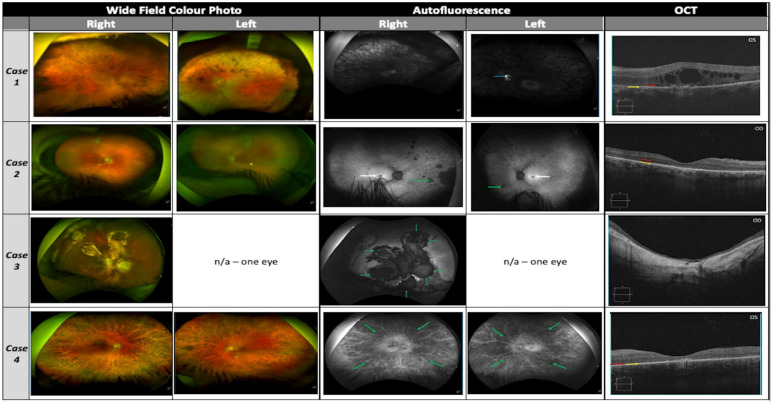
Diagnostic ophthalmic imaging for cases 1–4 with Optos ultra-widefield colour imaging, autofluorescence and OCT. Case 3 images available for right eye only due to corneal opacity. No images available for case 5.

VA was 6/36 (20/120) in the right eye (OD) and 6/60 (20/200) in the left eye (OS) with normal IOP. Horizontal corneal diameters were 12.0 mm bilaterally, with patent superior PI, and dense nuclear-sclerotic cataract OU. Fundus examination displayed peripheral intraretinal pigment migration, attenuated vasculature and pale crowded optic discs with optic disc drusen OS (Figure 3, case 1). AF displayed a grossly hypoautofluorescent picture bilaterally with hyperautofluorescent disc drusen OS (Figure 3, case 1, blue arrow). OCT demonstrated bilateral atrophy and disorganised architecture of the outer retina (Figure 3, case 1, red arrow: ONL remnant; yellow arrow: RPE; intervening layers absent/atrophic), with marked extensive cystoid macular lesions (CML)/foveoschisis (Figure 3, case 1). Nanophthalmos was diagnosed clinically with axial length measurements of 16.33 mm OD and 16.40 mm OS. Anterior chamber depths of OD 2.62 mm and OS 2.69 mm were recorded. Spherical equivalent refraction of OD +15.50 D and OS +15.75 D was in keeping with her axial lengths. Full field ERG was unrecordable, and consistent with a clinical picture of advanced rod-cone dystrophy. Only a handful of genes are linked to both nanophthalmos and RP, thus narrowing the potential genetic options (e.g., *MFRP*, *BEST1*, *CRB1*) [16].

Genetic testing, using Blueprint Genetics retinal dystrophy panel detected a homozygous pathogenic splice variant in *MFRP* [c.1124+1G>T]. *MFRP* encodes a frizzled-related protein which is involved in wingless related integration site signalling, a process implicated with both normal retinal function (post-natal) and ocular growth (embryologically and during childhood) [17,18]. This variant has been previously reported alongside another unrelated Irish patient with a consistent phenotype, together representing 0.2% of genotyped IRD pedigrees in Ireland [19]. Biallelic *MFRP* variants are associated with microphthalmia/nanophthalmia, RP, foveoshisis and optic disc drusen of variable severity (OMIM#611040) [20]. There is considerable diagnostic and clinical overlap in the terminology/definition of microphtalmia and nanophthalmia (i.e., axial length cut-offs likely representing a spectrum of disease severity based on the *MFRP* and other variants involved) [19,20]. Additionally, *MFRP*-related RP typically manifests in adulthood, without systemic/syndromic associations, consistent with this patient’s phenotype [19].

Resolution of this complex case by the OG-MDT resulted in a number of benefits for the patient. The patient underwent prompt bilateral cataract surgery, performed by a surgeon with experience handling complex IRD-related cataract procedures. The complexities of cataract surgery in nanophthalmic patients have been well documented [21]. Despite having no children, the AR nature *MFRP*-retinopathy allowed for genetic counselling of family members regarding carrier status and low risk of transmission to their offspring, though variants in *MFRP* and other loci have been implicated as modifiers of isolated hyperopia [18]. Finally, this diagnosis brought closure for the patient and ended the so-called ‘diagnostic odyssey’, of over 10 years, so familiar to patients with suspected IRDs [5,6,13].

### 3.2. Case 2: Syndromic IRD [Bardet-Biedl Syndrome (BBS)]

A 46-year-old male was referred with a clinical diagnosis of retinitis pigmentosa to the OG-MDT. The patient was given a clinical diagnosis of RP at age 37 years, with symptoms of nyctalopia since the second decade of life, followed by progressive visual field constriction and subsequent reduced VA (third decade of life). Clinical review by the OG-MDT revealed a diagnosis of type II diabetes mellitus at age 39 years, with self-reported difficulty controlling weight gain, resulting in increased body-mass-index. A history of hyperhidrosis was also reported. No other medical conditions of note were disclosed by the patient, and he denied a history of post-axial polydactyly. Initial family history gathering was positive for a sister with a clinical diagnosis of RP, and no consanguinity was reported in the pedigree. A visual acuity of OD 6/30 (20/100) and OS 6/38 (20/125) was recorded. He had bilateral early nuclear-sclerotic cataracts and normal IOP. Fundal assessment showed sparsely distributed peripheral intraretinal pigment migration circumferentially OU, with arteriolar attenuation and waxy pallor of the optic discs (Figure 3, case 2). There was no diabetic retinopathy. AF displayed foveal hypoautofluorescence with preserved isoautofluorescent parafoveal regions OU (Figure 3, case 2, white arrows). The peripheral retina displayed granular hypoautofluorescence with denser hypoautofluorescence in the nasal periphery bilaterally (Figure 3, case 2, green arrows). OCT revealed advanced bilateral retinal atrophic changes, with loss of outer retinal architecture/EZ and no evidence of CML (Figure 3, case 2, red arrow: ONL remnant; yellow arrow: RPE, no intervening structures). Visual field at presentation revealed maximal field of vision of ~30 degrees from fixation in each eye.

Panel-based NGS with a targeted retinal dystrophy panel of 351 genes (Blueprint Genetics), revealed a homozygous pathogenic variant in *BBS1* [c.1169T>G, p.(Met390Arg)] and no other likely candidate variants in IRD-associated genes. This is a well-documented disease-causing variant in *BBS1*, which is itself the most common BBS locus [22,23]. Interestingly this variant is associated with both syndromic and non-syndromic RP [24]. *BBS1* encodes one of a family of proteins (BBS-ome) required for ciliogenesis, more specifically of immotile cilia, defects in which can result in autosomal recessive (AR) BBS (OMIM#209900), RP (cone-rod dystrophy, 94%) with or without polydactyly, situs inversus obesity (89%), intellectual disability, kidney (52%), liver, and pancreas dysfunction of varying severity (diabetes 16%) [25,26]. BBS develops slowly through late childhood and early adulthood, with most patients diagnosed in this period [25]. BBS typically has a poor visual prognosis associated with early onset retinopathy [26]. However, within the spectrum of BBS causing variants, (Met390Arg) has been described as resulting in a milder phenotype [25,27].

Following this diagnosis, genetic counselling in discussion with the patient’s sister revealed a history of post-axial polydactyly for both the index patient (upper limb) and his sister (lower limb). Excision of supernumerary digits was performed in infancy, which the proband did not recall at the initial exam. This displays the importance of the genetic counsellors’ role once again in the collection of expanded family history/systemic phenotype.

The OG-MDT facilitated access to clinical genetics (genetic diagnostic tests), ophthalmology, dietetics (lifestyle modification for obesity), nephrology (monitoring of renal disease), and endocrinology (management of diabetes) to investigate and manage the constellation of systemic issues relating to BBS described above [25]. Quality and access of care for patients with multisystem genetic disease, such as BBS, may be significantly fragmented and the prevalence of syndromic IRD conditions is great enough to warrant dedicated multidisciplinary clinics (e.g., Usher syndrome 18% of RP and BBS 5% of RP) [28]. The BBS genetic diagnosis also allowed updated diabetic aetiology (in association with metabolic syndrome) rationalizing referral to a specialist endocrinologist [29]. AR inheritance was confirmed, in keeping with known BBS cases, and post-test genetic counselling of the low transmission risk to offspring was conveyed to the patient. Evidence surrounding the risk posed to carriers of pathogenic BBS variants (e.g., parents and children) is somewhat conflicting, However, there is a potential increased risk of obesity [25]. Management by the OG-MDT also allowed detection of the patient’s symptomatic sister, who will be offered genetic testing and review in the clinic.

### 3.3. Case 3: Early-Onset Severe Retinal Dystrophy (EOSRD) with Retinal Detachment [Knobloch Syndrome]

A 32-year-old female was referred to the OG-MDT with a past ocular history of congenital nystagmus, strabismus, high myopia, early onset cataract, pigmentary retinopathy, phenotypically consistent with EOSRD. This was complicated by bilateral rhegmatogenous retinal detachments for which the patient underwent multiple surgeries as well as bilateral vitreolensectomy with insertion of anterior chamber intraocular lenses (ACIOL) during childhood.

She reported no other medical history of note but commented on generalised hyperextensible joints. VA was OD 1/60 (20/1200) and OS no light perception with left exotropia. Slit lamp examination of the anterior segment revealed bilateral ACIOL with superior PI and dense band keratopathy precluding further examination OS. Intraocular pressure was within normal range in both eyes. Fundal examination OD revealed diffuse chorioretinal atrophic/fibrotic changes consistent with previous retinal detachment repair. She had geographic atrophy unmasking prominent choroidal vasculature affecting the foveomacular region OD (Figure 3, case 3). AF demonstrated a densely hypoautofluorescent appearance of the macula extending into the midperipheral retina (Figure 3, case 3, green arrows mark border of atrophy). OCT showed severe retinal thinning with loss of inner and outer retinal architecture with no clearly defined foveal pit or lamination (Figure 3, case 3). Axial length was 28.47 mm OD confirming axial myopia. During assessment the patient reported no history of developmental delay, skull abnormalities and displayed no stigmata of occipital encephalocele or repair. Family history confirmed a brother with a similar ophthalmic presentation.

The Blueprint Genetics Retinal Dystrophy panel revealed compound heterozygous pathogenic frameshift variants in *COL18A1* [c.2673dup, p.(Gly892Argfs*9) and c.3523_3524del, p.(Leu1175Valfs*72)]. Both are previously documented as pathogenic disease-causing variants [30,31]. *COL18A1* encodes type XVIII collagen, and pathogenic variants are associated with AR Knoblock syndrome (KS OMIM#267750) [32]. KS is characterised by high axial myopia, vitreoretinal degeneration, retinal detachment, macular atrophy, early onset cataract, and ectopia lentis, with or without occipital encephalocele [32,33]. Additionally, a wide spectrum of systemic features has been added to the expanded phenotype, including renal abnormalities and hyperextensible joints [32,33,34]. Female carriers of KS may provide a history of recurrent miscarriage due to aberrant brain development, which was not present in this case [32].

Identification of this variant and diagnosis of KS by the OG-MDT resulted in a number of positive outcomes for the patient and her family. Firstly, the patient underwent genetic counselling, allowing her to understand the cause for her ocular pathology, and its recessive inheritance pattern, allowing for future family planning. Secondly, it allowed identification of a brother with concerning history for similar pathology, who will now be assessed by the OG-MDT and offered genetic testing. Finally, the patient was put in contact with vision support and rehabilitation services to support her from a social and career perspective.

### 3.4. Case 4: Incidental Genetic Findings [Lynch Syndrome]

A 23-year-old female was referred to the OG-MDT with a clinical diagnosis of retinitis pigmentosa (RP). The patient was initially clinically diagnosed with RP in 2012 with symptoms of nyctalopia and photophobia, progressive VA reduction and visual field constriction. They reported no significant medical history and disclosed a family history including a maternal grandmother with breast cancer and two distant maternal relatives with an undiagnosed cause of sight loss [35]. VA was 6/15 (20/50) OD and 6/48 (20/160) OS in the right (OD). Anterior segments were unremarkable and IOP was within normal range. Posterior segment assessment showed dense intraretinal pigment migration circumferentially throughout the peripheral retina in both eyes (OU), with arteriolar attenuation and waxy pallor of the optic discs (Figure 3, case 4). AF displayed a diffusely hypoautofluorescent appearance to both fundi, with greatest density peripherally and relatively preserved isoautofluorescent para-foveal retina (Figure 3, case 4, green arrows: peripheral RPE atrophy). OCT revealed advanced bilateral retinal atrophic changes, with loss of outer retinal architecture/ellipsoid zone (EZ) and evidence of perifoveal CML most marked OD (Figure 3, case 4, red arrow: ONL remnant, yellow arrow: RPE, no remaining intervening laminae).

The patient was further assessed with a research grade 250-gene retinal dystrophy panel which did not detect any candidate causative variants. Genetic diagnosis rate using this panel [15], has been approximately 70% in the total Target 5000 cohort [15]. After discussion at the OG-MDT, trio WES (i.e., proband and parents) was conducted in an attempt to resolve the pedigree (Blueprint Genetics, whole exome family). Unfortunately, WES also failed to resolve the patients phenotype; however, an incidental pathogenic *PMS2* [c.137G>T, p.(Ser46Ile)] variant was detected in both the proband and her mother [35].

*PMS2* encodes a crucial component of the DNA mismatch repair facility, and pathogenic variants in this gene are associated with autosomal dominant (AD) Lynch syndrome (OMIM#614337) [36]. Lynch syndrome is a predisposition to colorectal and endometrial cancer and is the most common heritable colorectal cancer syndrome (3% of all colon cancer diagnoses) [36]. Furthermore c.137G>T, p.(Ser46Ile) is a well-documented founder variant associated with Lynch Syndrome [36]. The disclosure of incidental findings, particularly of the life-altering variety represent an ethical conundrum, with increasing likelihood of secondary and incidental findings as the use of WES and WGS become more prevalent [37,38]. Secondary findings have been shown to arise in 1.7% of WES tests and the importance of pre-test genetic counselling and informed patient consent regarding the optional disclosure of such findings has been highlighted [38,39]. The American College for Medical Genetics and Genomics (ACMG) provides an updated list of important incidental and secondary genetic findings which should be reported to those affected [40]. Prior to WES, the patient consented to disclosure of incidental findings signing the Blueprint Genetics consent form for WES and underwent pre-test genetic counselling. The result and need to disclose was discussed and agreement on that was made by MDT team. Following the disclosure of the *PMS2* variant to the patient and her mother, comprehensive genetic counselling was provided, which elucidated a more extensive family history of cancer [35]. This case highlights the crucial role of the genetic counsellor in the OG-MDT, providing pre-test counselling prior to WES, and comprehensive post-test counselling regarding the diagnosis of a potentially life-altering incidental finding. Identifying this cancer predisposition resulted in referral to colorectal screening services for both the patient and her mother to facilitate detection of potential malignancy in line with current clinical guidelines [41]. The mother went on to have prophylactic surgery to reduce the risk of cancer-related mortality. Familial variant testing was offered to maternal relatives confirming the pathogenic *PMS2* variant in additional family members. The patient will now continue on the Target 5000 OG-MDT gene negative diagnostic pathway (i.e., trio WGS, array CGH), should she desire, in an ongoing attempt to resolve her phenotype through further genetic testing [42].

### 3.5. Case 5: Paediatric Syndromic Ophthalmic Neurodegeneration [Batten Disease]

A 5-year-old female was referred with severe retinal dystrophy and clinical findings suggestive of macular dystrophy. Her presenting symptoms included decreased peripheral awareness, with noted clumsiness and related accidental injury, followed by a rapid deterioration in visual function over a one-year period. She was otherwise healthy with no known family history of IRD; however, family history was positive for a sister with trisomy 21, and mother with high myopia and non-progressive hearing loss of unknown etiology. The VA was 6/190 (20/630) bilaterally. Dilated examination revealed clear crystalline lenses, bilateral bullseye maculopathy and peripheral intraretinal pigment migration with atrophic changes. There was no nystagmus present. Full field electroretinogram demonstrated no definite scotopic or photopic response, with results suggestive of significant cone-rod dystrophy. Clinical imaging was not available for presentation in this case.

Molecular genetic testing using the Blueprint Genetics 351 gene IRD panel identified a pathogenic homozygous deletion in *CLN3,* [c.(460+1_461-1)_(677+1_678-1)del], encompassing exon 8 and 9 of *CLN3*, refining the diagnosis to Batten disease (OMIM#204200) [43]. *CLN3* encodes an ATP synthase chaperone [43], failure of which leads to premature photoreceptor degeneration and CNS neuronal apoptosis, with deposition of autofluorescent lipopigments in neuronal cells. The main variant is the 1 kb deletion seen in this case [44]. Batten disease, also known as juvenile neuronal ceroid lipofuscinosis is an autosomal recessive condition which, in addition to a retinal dystrophy of onset between 5 and 10 years of age, subsequently results in development of sensory, motor or psychiatric manifestations. Seizures and intellectual decline are a prominent feature of the disease, with a high mortality in the second to third decade of life [43].

Seven months later, the patient was admitted under the paediatric neurology team for uncontrolled seizures. Having a genetic diagnosis helped to swiftly identify the underlying seizure aetiology, optimize seizure management, and enable genetic counselling for the patient/family. Although this is a rare condition, with a prevalence of 1/25,000 births [43], visual features precede systemic features in Batten disease, making the ophthalmologist a likely first point of contact. Empathetic genetic counselling was of upmost importance in this case, as the diagnosis of Batten disease carries such profound consequences for the patient, and their family. Having an established OG-MDT is critical for facilitating early diagnosis, with prompt involvement of paediatric neurology and genetic counselling in such cases. Pre-clinical studies have shown promising disease modification from early systemic gene therapy for *CLN3* giving hope for this devastating disease and highlighting the importance of early genetic diagnosis [45].

## 4. Discussion

### 4.1. Justification for an Ocular Genetic Service

The aforementioned cases illustrate the benefits of a coordinated OG-MDT and justify including this service in centres seeing patients with IRD and other rare heritable eye conditions. Without the central coordination and collaborative approach, this high level of diagnostic and therapeutic harmony would not be possible [5,9,12]. Collaborative team expertise, such as those capitalised upon and developed within a structured patient-specific MDT framework, can achieve results beyond that of the individual, while evolving new knowledge and skills in the process [46]. As IRDs are rare diseases, concentrating care in single centres rather than dispersing throughout general ophthalmology clinics allows access to specialised clinical and genetic testing modalities (funding and appropriate laboratory staff) [12]. The higher case load of rare diseases allows ongoing improvement of expertise, team learning and research discovery.

A core team of ophthalmologists/retina specialists, molecular and clinical geneticists, and genetic counsellors is required to resolve and manage complex IRD cases. Additional experts (e.g., neurology, otorhinolaryngology, nephrology, pulmonology, etc.) can be involved for syndromic cases, which out of efficiency, may be coordinated into focused syndromic IRD OG-MDT meetings. This is a commitment for all involved, and use of a standardized template for case reporting and actionable outcomes can facilitate efficiency and remove the need for redundant consultations, which are resource-consuming and a burden for the patient.

### 4.2. The Benefits for Patients from the OG-MDT Include

1. Clinical management/visual rehabilitation.
  a. Immediate treatments (e.g., refraction, cataract surgery, treatment of cystoid macular lesions);
  b. Delayed (e.g., novel disease-modifying therapies such as gene therapy).
2. Diagnostic certainty -confirmation of a genetic aetiology can often link many symptoms together (e.g., diabetes, polydactyly and cone-rod dystrophy in BBS). This can be a source of great relief, ending the long uncertainty regarding diagnosis.
3. Genetic Counselling—empowered with new genetic data, many life decisions can be facilitated, including:
  a. Family planning—understanding the inheritance pattern associated with a given genotype can inform both family planning and screening of relatives for ocular or systemic effects.
  b. Career and educational planning can be supported appropriately, enabling people with IRD to continue the enjoyment of good quality of life.
4. Incidental and secondary findings.

In the course of genetic testing, other genetic features may be identified, either related to the ophthalmic phenotype or to unrelated conditions (e.g., cardiac or cancer risk alleles). This may then open options to modify outcomes and provide a chance to pre-empt life-changing health concerns.

### 4.3. Benefits for the Vision Science Community

Using a multi-specialty approach, novel phenotype–genotype associations can be elicited. Publications of such findings may aid in diagnosis of similar cases internationally and help inform novel treatments (e.g., BBS-associated diabetes/obesity). Collection of rare disease cases in a single centre of excellence can enable observational (e.g., natural history studies) and interventional (e.g., neuroprotectant, subretinal gene therapy) studies with greater impact than disseminated isolated centres with low IRD case volume [47,48,49]. Large databases of well characterized IRD cohorts enable international collaborative projects which will benefit both scientific understanding and patient quality of life.

This concept can be introduced in other centres, which can be linked to central patient/clinical support resources such as the European Reference Network for Rare Eye Disease (ERN-EYE) and the Foundation Fighting Blindness in the USA. Though there are challenges in recruiting interest and participation from the various specialty members, a core team with expertise in ophthalmic genetics can gradually expand their role and demonstrate value to the clinical care of patients with rare and complex genetic conditions.

Roadblocks to incorporation of the OG-MDT model are (1) funding required to have access to the relevant specialists on a regularly scheduled basis, and for clinical and genetic investigations, (2) adequacy of referral base of appropriate ophthalmic genetic cases and (3) expertise available at the clinical centre, which may be overcome by collaboration and virtual meetings between allied centres. The cost of genetic testing is ever reducing and the efficiency/scope is increasing (range: small panel EUR 550; large panel EUR 870), with some charitably funded initiative providing free genetic testing for IRD patients.

In summary, this paper demonstrates the benefits of an OG-MDT to patients with rare heritable eye diseases including IRDs. Facilitating the holistic resolution of complex and syndromic cases the structured OG-MDT provides additional benefits by developing team member expertise and furnishing the wider ophthalmic genetics community with a wealth of clinical and genetic information which can improve diagnosis and management of IRD. This format can be adopted/developed by similar centres around the world, bringing with it the myriad benefits discussed herein.

## Data Availability

All data generated or analysed during this study are included in this article. Further enquiries can be directed to the corresponding author. Anonymized source data can be shared upon reasonable request.
